# Ageing transitions in a network of Rulkov neurons

**DOI:** 10.1038/s41598-021-03844-1

**Published:** 2022-01-10

**Authors:** Dhrubajyoti Biswas, Sayan Gupta

**Affiliations:** 1 Department of Physics, Indian Institute of Technology Madras, Chennai, Tamil Nadu 700036 India; 2 The Uncertainty Lab, Department of Applied Mechanics, Indian Institute of Technology Madras, Chennai, Tamil Nadu 700036 India; 3 Complex Systems and Dynamics Group, Indian Institute of Technology Madras, Chennai, Tamil Nadu 700036 India

**Keywords:** Computational science, Dynamical systems

## Abstract

The phenomenon of ageing transitions (AT) in a Erdős–Rényi network of coupled Rulkov neurons is studied with respect to parameters modelling network connectivity, coupling strength and the fractional ratio of inactive neurons in the network. A general mean field coupling is proposed to model the neuronal interactions. A standard order parameter is defined for quantifying the network dynamics. Investigations are undertaken for both the noise free network as well as stochastic networks, where the interneuronal coupling strength is assumed to be superimposed with additive noise. The existence of both smooth and explosive AT are observed in the parameter space for both the noise free and the stochastic networks. The effects of noise on AT are investigated and are found to play a constructive role in mitigating the effects of inactive neurons and reducing the parameter regime in which explosive AT is observed.

## Introduction

Investigating the dynamical behaviour of an animal brain, formed as an intricate web of large number of interconnected neurons^1^, requires modelling it as a complex dynamical system with underlying network structure^2–4^. Here, each neuron is a dynamical entity. A complex dynamical system approach enables investigating the time evolution of a large number of interconnected subsystems, the dynamics of each of which are reasonably well understood in isolation but whose collective dynamical behaviour can be significantly different due to the interaction effects. The complexity arises from the interactions, on account of the coupling between the subsystems. Each subsystem is modelled as a node and the mutual interactions due to coupling are modelled as links/edges forming a complex network. One of the most widely investigated complex networked system is the Kuramoto model of phase oscillators^5,6^. The scale of such complex networked systems can range from 10 to 20 in “low” dimensional oscillator networks^7^ to billions of interacting neurons, for example in the human brain model^8^. Phenomena like synchronization^9–11^, explosive synchronization^12–14^ and explosive death^15,16^ are the hallmarks of such complex systems and have been studied across domains ranging from networks of neurons^17^, mechanical oscillators^18^ to chemical systems^19^.

Investigating the capability of a networked system to operate dynamically even when segments of the system malfunctions is of interest to estimate its robustness to external and/or internal perturbations/malfunctions. Such robustness is observed in many biological systems—such as the brain^1^—which continue to function, maybe sub-optimally, after trauma. In this context, one of the more interesting recent developments has been to study Ageing Transitions (AT) in networks of dynamical systems. In ageing neuronal networks, some nodes (neurons) lose their functionality and become inactive dynamically. When the fraction of inactive nodes is low, the networked system might be able to compensate the loss through coupling and continue to exhibit “active” dynamical behaviour. However, as the fraction of inactive nodes increase, the system collectively *transitions* to a state where there is no significant “active” dynamical behaviour in the system. AT was first observed in Stuart-Landau (SL) oscillators^20^ and later in many systems like globally coupled networks^21,22^, locally coupled oscillators^23^, fractional order SL oscillators^24^, Hindmarsh-Rose neurons^25–27^ and time-delayed interaction systems^28^ among others. The nature of AT from the active state of the network to the inactive state can be gradual, monotonic or explosive and depends on several key parameters associated with the problem. A sudden or explosive transition is characterised by a sharp change in the system’s dynamical characteristics on a slight change of one of the parameters. These type of transitions are potentially detrimental as this leads to a total loss of capability of the network, without prior warning. There is therefore a need to investigate the parameter regimes at which these explosive transitions occur. An order parameter, which is a measure of the collective dynamical characteristic of a complex network in which the individual components can behave quite differently, is defined to qualitatively and quantitatively investigate the parameter regimes at which these ageing transitions are observed.

This article focuses on investigating the phenomenon of AT in a network of Rulkov neurons, with the fraction of “inactive” neurons as one of the important parameters. As is well known, neuronal dynamics is typically characterized as a discontinuous system comprising of two distinct states—a silent or inactive state and a spiking or active state. The Rulkov neuron is a simple map based model which captures the discontinuous nature of the neuronal dynamics and unlike differential equation based models, enables numerical studies at significantly cheaper computational costs. This enables carrying out parametric studies for networked systems with a large number of Rulkov neurons. A mean field coupling model is proposed for the neuronal interconnections and the conditions for AT are investigated using measures based on standard order parameters. Subsequently, the dynamics of the networked system is investigated when the mean field coupling model is modified to be stochastic. Parametric studies on the stochastic system reveal that noise could play a constructive role in altering the dynamical characteristics of the network by delaying the onset and the nature of AT. In investigating the dynamics of ageing systems for a given set of parameters, the fraction of “inactive” nodes are taken to be constant as the time-scale of ageing is usually several orders of magnitude larger than the time-scale of the system dynamics.

## Mathematical model

The Rulkov map^29,30^ involves two state variables $x_n$ and $y_n$, where the former represents the membrane potential of the neuron and is the primary quantity of interest while the latter is an internal variable necessary to capture the dynamics; see Eqs. (1–3). The suffix “*n*” represents the *n*-th discrete time step. Depending on the choice of the parameters, a solitary neuron exhibits three distinct long-time dynamical states^29^: dead/silent (DS), tonal spiking (TS) and spiking bursting (SB). In this study, the parameters are selected such that the individual neurons exhibit either the DS or TS states. In the case of networked neurons, the map consists of additional terms (see Eqs. (4–8)) that take into account the coupling effects between the *i*-th and *j*-th neuron of the network. A symmetric mean field coupling model is adopted where the strength of the coupling is proportional to the mean of the differences between the membrane potential of the *i*-th neuron and the corresponding membrane potential of all other neurons with which it is directly connected; see Eqs. (6a–6b). This implies that depending on the instantaneous values of the membrane potentials, the nature of the coupling between any two neurons could be either inhibitory or excitatory. The coupling proportionality constant $g_{ij,n}$ is assumed to be time dependent comprising of a constant mean term $g_m$ denoting the average coupling strength and an additive fluctuating component, modelled as Gaussian white noise. The fluctuating component accounts for the time dependent modelling uncertainties in the coupling, with the intensity of the fluctuations being represented by *D*. The network is in the form of a random coupled map lattice (CML)^31^, with each node (neuron) having a connection probability $P_n$. Small values of $P_n$ lead to a sparsely connected network, while higher values implies a highly connected or dense network. The probability that a particular neuron is inactive (or dead) is defined using parameter $P_d$. For larger values of $P_d$, it is expected that the number of inactive neurons in the network increases, implying an increase in the “age” of the network. The network dynamics is governed by an intricate interplay between the four parameters $g_m$, *D*, $P_d$ and $P_n$. To quantify the collective dynamical characteristics of the network, an order parameter *A* is defined, which is a normalised measure for the largest amplitude of the oscillations in the steady state condition averaged across all neurons in the network; see Eqs. (10–11). *A* can take values between unity and zero, with zero indicating that none of the neurons are oscillating and hence the network collectively becomes inactive (or dead).

## Results

Exhaustive parametric investigations have been carried out numerically. The representative interesting results that bring out the salient features for both the noise-free and the stochastic cases are discussed next.

### Noise free system

**Figure 1:**
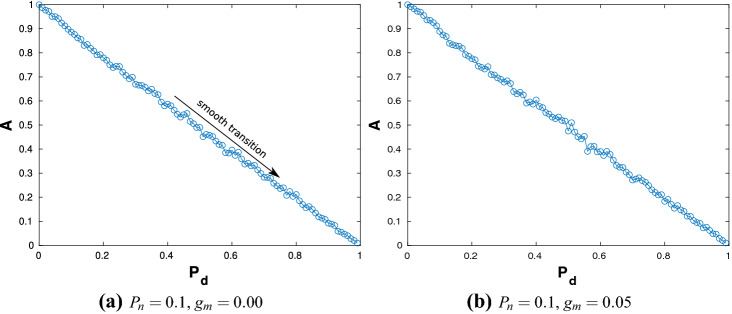
The order parameter *A* plotted as a function of $P_{d}$ for $P_{n}=0.1$ and (**a**) $g_{m}=0.0$ and (**b**) $g_{m}=0.05$ exhibiting *smooth* AT.

The noise free system is when the intensity $D=0$. The collective dynamics is quantified in terms of *A* as a function of $P_d$ for different values of $g_m$ and $P_n$. Figure 1 shows the variation of *A* with respect to $P_{d}$, for a sparse ER network with $P_{n}=0.1$. Figure 1a,b consider the cases when $g_{m}=0.0$ and $g_{m}=0.05$ respectively. The former case essentially considers a set of uncoupled neurons and the latter represents very weak coupling in an already sparse network. In both cases, *A* is observed to decrease linearly with $P_{d}$. This is expected as with increasing values of $P_d$, the number of inactive neurons increases, thereby bringing down the value of the averaged normalised order parameter. The zero coupling or low coupling strength implies that the active neurons have little effect on the network. In both cases, $A \rightarrow 0$ as $P_d \rightarrow 1$.

**Figure 2:**
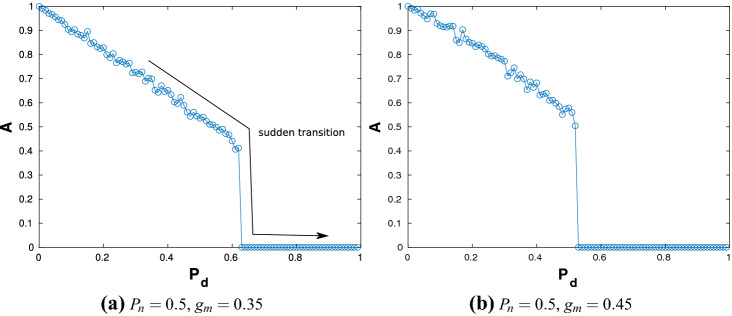
The order parameter *A* plotted as a function of $P_{d}$ for $P_{n}=0.5$ and (**a**) $g_{m}=0.35$ and (**b**) $g_{m}=0.45$ exhibiting *sudden* AT.

Next, simulations are carried out for an ER network with $P_n=0.5$, which represents a more connected network. The variation of *A* with respect to $P_d$ is shown for $g_m=0.35$ in Fig. 2a and for $g_m=0.45$ in Fig. 2b.

These $g_m$ values indicate a higher coupling strength. These figures reveal that there is a sudden drop to zero in the value of *A* at a particular value of $P_d$ indicating a sudden transition of the network to the DS.

**Figure 3:**
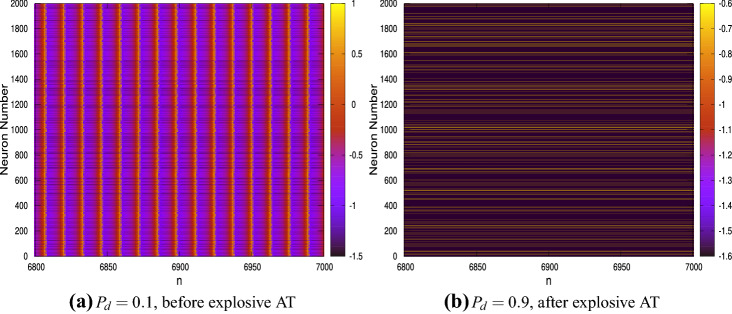
Time history diagrams showing the dynamical behaviour of each of the $N=2\times 10^3$ neurons (plotted on the ordinate) with time/iterate (plotted on the abscissa for $n= 6800$ to $n=7000$); (**a**) the dynamics for the system before AT ($P_{n}=0.5$, $g_{m}=0.35$ and $P_{d}=0.1$); (**b**) the dynamics for the system after AT ($P_{n}=0.5$, $g_{m}=0.35$ and $P_{d}=0.9$).

This sudden discontinuous transition in the state of the network is defined as explosive AT. To gain insights into the collective dynamics of the network, raster plots which show the time histories of the dynamics of each neuron are investigated; see Fig. 3. Here the abscissa is iterate *n*, the ordinate is the neuron number *i* and the colour code denotes the values of the state variable $x_{n}$ for each neuron in the network. Figure 3a shows the plots for the case $P_{n}=0.5$, $g_{m}=0.35$ and $P_{d}=0.1$ for $n=$ 6800–7000. The choice of $P_d$ indicates that the regime is to the left of the explosive AT seen in Fig. 2. A homogeneous banded figure pattern is observed, with the bands appearing vertically, indicating that almost all the neurons undergo spiking synchronously. A closer inspection reveals that in between there are neurons which do not undergo any oscillations and these appear as horizontal lines. These are the inactive neurons. The numerical values of $x_n$ of these inactive neurons is not zero; in fact, they have different values at the DS which remain constant for all iterates indicating a lack of dynamics. A similar plot for the network having identical parameters but with $P_d=0.9$—which is in the regime to the right of the explosive AT—is shown in Fig. 3b. Here, one can see that the figure comprises of a set of parallel lines indicating that none of the neurons exhibit spiking. All the neurons are therefore in the DS state, and though they have different values of $x_n$, the order parameter *A* by its definition is zero. It is important to note that as the number of inactive neurons increase in the network, the effects of the active neurons on the network dynamics diminishes and beyond a critical threshold value of $P_d$, the higher coupling strength of a reasonably connected ER network ensures that the effects of the active neurons die down with time and the entire network exhibits a DS. Note that Fig. 3a shows the time histories only after the effects of the transients have died down. Also, unlike in other systems associated with explosive transitions, hysteresis is not observed here.

**Figure 4:**
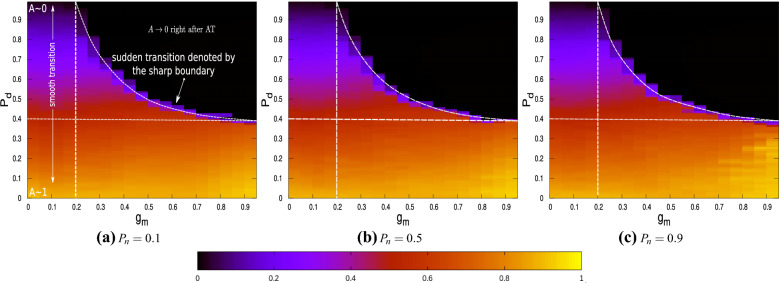
The order parameter *A* plotted as a function of both $P_{d}$ and $g_{m}$ for (**a**) $P_{n}=0.1$, (**b**) $P_{n}=0.5$ and (**c**) $P_{n}=0.9$. The dark/black coloured region shows where $A\rightarrow 0$ whereas yellow shows where $A\rightarrow 1$. The colour-map which is common for all three figures is shown below the plots.

A parametric study is carried out to investigate in detail the behaviour of the network as $P_d$, $g_m$ and $P_n$ are varied. Figure 4a–c show the contours of the order parameter *A* as a function of $P_d$ and $g_m$ for three different values of $P_n$, corresponding to network topologies that are sparse, intermediate and dense.

The colour-map is same for all cases, with yellow denoting $A\rightarrow 1$ and black denoting $A\rightarrow 0$. The black region in these figures denote the parameter regime where the network is globally in the DS. An explosive AT is observed where the colour gradients are sharp. The sharp boundary, in all three cases, indicates the boundary of explosive AT in the parameter space with the colour map indicating the size of the explosive transition. It is interesting to note that the figures look qualitatively similar in all the three cases, indicating that the collective network behaviour is universal irrespective of the topology of the ER network. It is seen that irrespective of the ER network topology or the coupling strength, the critical threshold value for $P_d$ required to observe AT is approximately 0.4. This is shown by the horizontal dotted lines in these figures. The vertical dotted lines demarcate the parameter regime such that the region left of this line exhibits smooth transition and to the right shows explosive AT. It is therefore seen that $g_m$ should be at least 0.2 for explosive AT to occur, irrespective of the ER network topology.

**Figure 5:**
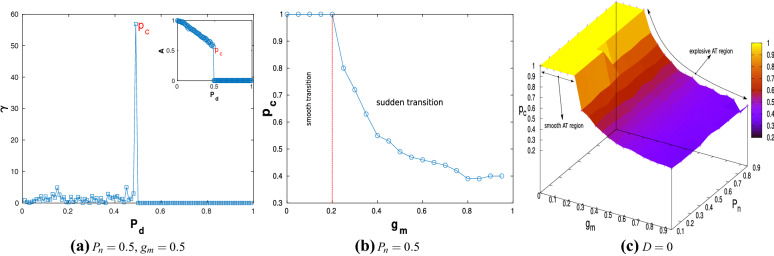
(**a**) Variation of $\gamma$ with $P_{d}$; a peak is observed when explosive AT occurs (inset shows the corresponding variation of *A* with $P_d$). (**b**) Variation of $p_{c}$ with $g_{m}$ for $P_{n}=0.5$. (**c**) Global variation of $p_{c}$ with $P_{n}$ and $g_{m}$.

The *critical* “death” or “inactivation” probability for $P_d$, denoted by $p_{c}$, beyond which the network dynamics explosively transitions to a global DS can be quantitatively estimated through the gradient measure $\gamma =\big |\Delta A/\Delta P_{d}\big |$. As is obvious, $\gamma$ shows a high value when there is a discontinuous jump in *A* as $P_d$ is changed. Figure 5a shows the variation of $\gamma$ with $P_{d}$ for the case $P_{n}=0.5$, $g_{m}=0.5$.

From the spike, $p_c$ is identified to be 0.5 and this can be corroborated from the inset in Fig. 5a. It is interesting to note that the values of $\gamma$ left of the spike are small but exhibit irregular fluctuations, while the values are identically equal to zero to the right of the spike. This indicates that for $P_d > p_c$, the network has globally attained the DS. The variation of $p_c$ with $g_m$ for the same topology of the ER network is shown in Fig. 5b. The sudden transition in the nature of the curve for $g_m >0.2$ clearly demarcates the smooth and explosive transition regimes. It is also observed that $p_c$ does not take values less than 0.4 confirming the earlier observation that explosive AT is not observed for $P_d <0.4$. The monotonically decreasing nature of the curve underlines the fact that with an increase in the coupling strength and in turn a stronger coupling of the constituents in the network, the global DS of the network is achieved with lower $P_d$. This seems to imply that the DS is a stronger attractor than the TS. Figure 5c shows the variation of $p_c$ with respect to both $g_m$ and $P_n$ confirming that these observations are irrespective of the topology of the ER network.

### Stochastic system

Stochasticity is introduced in the system through additive noise to the mean coupling strength $g_{m}$ by considering $D\ne 0$ in Eq. (8). The noise is modelled as a discretized Gaussian white noise process such that the additive noise at each iterate $\zeta _{n}$ is standard normal and ${\mathbb {E}}[\zeta _{n} \zeta _{m}]=\delta _{nm}$ where $\delta _{nm}$ is the Kronecker delta. Here, ${\mathbb {E}}[\,\cdot \,]$ is the expectation operator. As ${\mathbb {E}}[g_{ij,n}]=g_m$, the noise does not affect the average coupling strength in the ER network. Though the noise is additive with respect to $g_{m}$, it enters the system as multiplicative noise as $g_{ij,n}$ is a system parameter.

**Figure 6:**
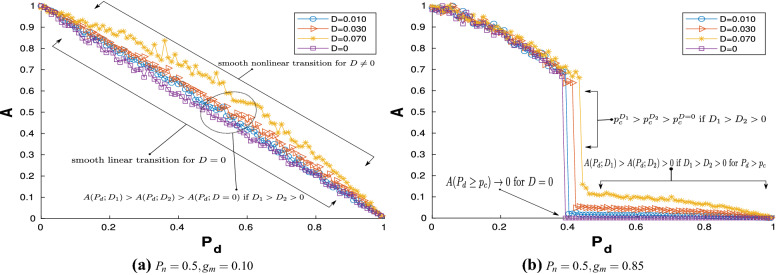
Variation of *A* with $P_{d}$; $P_{n}=0.5$ and $D=0.01,0.03,0.07$: (**a**) $g_{m}=0.1$—smooth transition is observed; (**b**) $g_{m}=0.85$—explosive AT is observed.

Figure 6 presents the variation of *A* as a function of $P_{d}$, when $P_{n}=0.5$ for the stochastic network for (a) $g_{m}=0.1$ and (b) $g_m =0.85$.

The corresponding variation for the noise-free network ($D=0$) is also shown in the same figures for comparison. A smooth transition is observed in Fig. 6a, where $A \rightarrow 0$ as $P_d \rightarrow 1$. An important observation is that *A* for the stochastic network is greater in comparison to the noise free system for almost the entire regime indicating that the amplitude of the oscillations, on an average, increase with noise intensity *D*. Explosive AT is observed for higher values of the mean coupling strength for both the noise free as well as the stochastic case as can be seen in Fig. 6b. The stochastic network however does not exhibit global DS behaviour post explosive AT, unlike the noise free network. As characterised by the measure *A*, as $P_d$ is further increased beyond $p_c$, there is a steady decline in the oscillations and a global DS is attained as $P_d\rightarrow 1$. The magnitude of the oscillations post explosive AT are higher for larger noise intensities.

**Figure 7:**
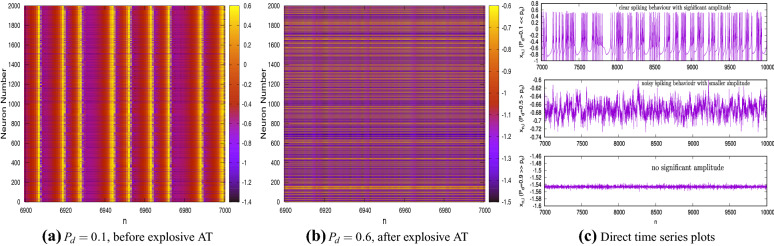
Time series diagrams showing the dynamical behaviour of each of the $N=2000$ neurons (plotted on the ordinate) with time (plotted on the abscissa); (**a**) Dynamics for the system before AT ($P_{n}=0.5$, $g_{m}=0.85$ and $P_{d}=0.1$). (**b**) Dynamics for the system after AT ($P_{n}=0.5$, $g_{m}=0.85$ and $P_{d}=0.6$). (**c**) dynamics of randomly selected neurons for different values of $P_{d}$. In all cases, $D=0.05$, while $P_{n}=0.5$ and $g_{m}=0.85$.

An important observation from Fig. 6b is that noise delays the onset of the explosive AT. This is clearly evident from the figure where the explosive AT for $D=0$ is observed to be at $P_{d}\approx 0.4$, while as *D* becomes larger, explosive AT occurs at higher values of $P_d$. To gain further insights into the collective dynamics of the network, the time histories of the membrane potential $x_{i,n}$ for each neuron is investigated through raster plots; see Fig. 7a–b. Figure 7a shows the plot for $D=0.05$, $P_{n}=0.5$, $g_{m}=0.85$, $P_{d}=0.1$ and for $n=$ 6800–7000 and denotes a regime prior to explosive AT. Note that the raster plots are shown only for a small segment in time after the initial transients have died down. The banded pattern observed is similar to Fig. 3a, indicating that even for the stochastic network all the active neurons exhibit synchronous spiking. The horizontal lines are the inactive neurons having a constant value of $x_{i,n}$ for all *n*; these values are however different for each of the inactive neurons. However, unlike the noise free network, the vertical bands are not equally spaced indicating that the spikings are no longer regular for the neurons exhibiting spiking. A similar raster plot for the stochastic network with identical parameters but for $P_{d}=0.6$, is shown in Fig. 7b. As observed in Fig. 6b, this corresponds to post explosive AT regime but the network does not attain global DS. A close inspection of Fig. 7b reveals that while the horizontal lines are predominant and therefore indicative of a large number of neurons in DS, vertical bands are also observed; this is in contrast to the observations in Fig. 3b. The blue-black vertical banded pattern interspersed among the horizontal lines indicates that some neurons are still exhibiting oscillations and which are synchronous. This explains the positive values of *A* post explosive AT for the $D\ne 0$ case in Fig. 6b. These inferences are further verified from the time series plots for randomly chosen active neurons shown in Fig. 7c. The topmost panel shows the dynamics of a neuron before explosive AT ($P_{d}< p_{c}$), the middle panel shows the dynamics just after explosive AT ($P_{d}= p_{c} +\epsilon$ where $\epsilon >0$ is a small number) and the bottom panel shows the dynamics for $P_d$ significantly greater than $p_c$ and almost close to unity. The oscillations for the post explosive AT regime are observed to be inherently noisy whose amplitude as well as the mean value decrease with an increase in $P_d$.

**Figure 8:**
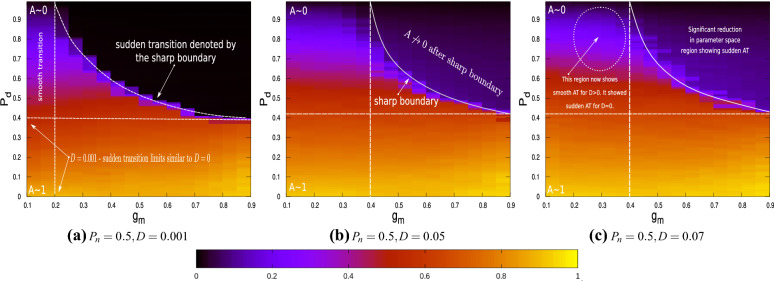
The order parameter *A* plotted as a function of $P_{d}$ and $g_{m}$ for $P_{n}=0.5$ and (**a**) $D=0.001$, (**b**) $D=0.05$ and (**c**) $D=0.07$. The dark/black coloured region shows where $A\rightarrow 0$ whereas yellow shows where $A\rightarrow 1$. The colour-map which is common for all three figures is shown below the plots.

A parametric study is next carried out to investigate the behaviour of the stochastic network as $P_{d}$, $g_{m}$ and *D* are varied. Figure 8 shows the contour map for *A* in the $g_m - P_d$ parameter space for three different cases of noise intensities. The colour-map scale is same for all cases, with yellow denoting $A\rightarrow 1$ and black denoting $A\rightarrow 0$. For $D=0.001$, a sharp boundary for the black region is observed in Fig. 8a indicating explosive AT and that *A* is (or close to) zero in the regime post explosive AT. This figure is similar to Fig. 4; the bounds on $g_{m}$ and $P_{d}$ for which the sharp boundary is encountered are identical. The contour maps shown in Fig. 8b,c, for $D=0.05$ and$D=0.07$ respectively, are however qualitatively different. These figures still show a sharp boundary in the $g_m-P_d$ space, indicating explosive AT, but post explosive AT, the order parameter *A* does not go to zero. An inspection of Fig. 8 reveals that no explosive AT is observed for $P_d <0.4$ for the entire parameter regime. However, as *D* increases, the minimum values for $g_m$ at which explosive AT is observed, increases. A comparison of Fig. 8a,c reveal that the regime in the range of $0.2< g_m <0.4$ which showed explosive AT for low values of *D*, exhibit smooth AT as *D* increases. This implies that multiplicative noise can not only increase the region in the $g_m -P_d$ parameter space at which dynamic neuronal activities are observed, explosive transitions which result in sudden loss of the functionality of the system is also impeded.

**Figure 9:**
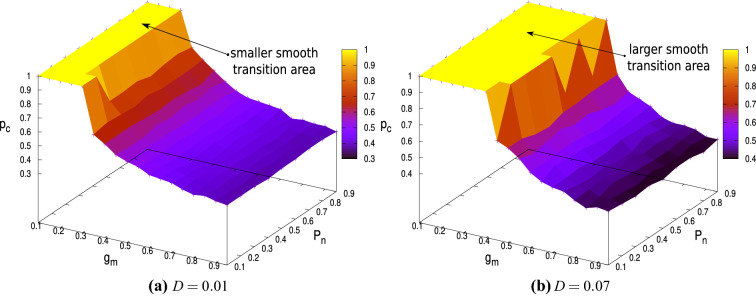
The critical inactivation probability $p_{c}$ plotted as a function of $g_{m}$ and $P_{n}$ for (**a**) $D=0.01$ and (**b**) $D=0.07$.

These observations are true for all network connectivities $P_{n}$ as has been shown in Fig. 9, where the variation of critical inactivation probability $p_{c}$ is shown in the $g_{m}-P_{n}$ parameter space for $D=0.01$ (Fig. 9a) and $D=0.07$ (Fig. 9b). These two cases represents intermediate and high noise intensity.

An additional case that has been studied is to investigate the effects of inhomogeneity associated with each neuron. This was carried out by considering the parameter $\sigma _i$, which has been so far assumed to be identical for all neurons, to have small variations about the mean value $\sigma$. Mathematically, this is modelled by adding a small random perturbation $\chi \xi _i$ to the *i*th neuron, where $\xi _i$ is sampled from a standard normal distribution and $\chi$ is the strength of the perturbation. Simulations were carried out for $\chi =0.01$, and as $\sigma$ is taken to be 0.6, the variability in $\sigma _i$ ranged from 0.57 to 0.63 with 99.99% probability. Numerical simulations revealed no appreciable qualitative or quantitative differences in the results. This can be explained by the fact that small changes in $\sigma$ are not strong enough to enable a transition of the Rulkov neuron from DS to TS or vice versa. Considering higher values of $\chi$ that enable such transitions would imply that $P_d$ can no longer be treated as an independent parameter in the model and one would need to instead consider $\chi$ as the independent parameter. Instead, considering $P_d$ as the independent parameter enables taking into account implicitly the larger variabilities in the parameters associated with a neuron.

## Discussion

The phenomenon of explosive AT in an ER network of coupled Rulkov neurons has been studied, with network connectivity, coupling strength and the fractional ratio of inactive neurons being the parameters of interest. This has been investigated for both the noise free network as well as the stochastic network. In the latter case, the mean inter-neuronal coupling strength have been superimposed with Gaussian white noise at each iterate. An order parameter has been defined for quantifying the dynamical state of the network. It has been shown that explosive AT occurs only when at least $40\%$ of the neurons become inactive both for the noise free and the noisy network, and also depends on the mean coupling strength. For the noisy network, it has been shown the regime at which explosive AT occurs in the parameter space reduces with increasing noise intensity. Moreover, increasing the noise intensity prevents the network from going to DS. However, while noise appears to play a constructive role in preventing explosive AT in certain regimes, the neuronal dynamics in regimes post explosive AT even though do not attain DS, they are noisy and irregular unlike the regular spiking nature expected of neurons. The observations from this study can pave the way for more detailed analysis for probable treatments of impaired neuronal networks.

## Methods

### Single neuron

The Rulkov map^29,30^, involving two state variables $x_n$ and $y_n$, is given by

1 $$\begin{aligned} x_{n+1}&=f_{\alpha }(x_{n},y_{n}+\beta _{n}), \end{aligned}$$

2 $$\begin{aligned} y_{n+1}&=y_{n}-\mu (x_{n}+1)+\mu \sigma _{n}. \end{aligned}$$

Here, $x_{n}$ represents the membrane potential of the neuron and is the primary quantity of interest. The variable $y_{n}$ does not have any particular biological connotation but is important to capture the neuronal dynamics and therefore can be thought of as an “internal” variable. The suffix “*n*” represents the *n*-th discrete time step. $f_{\alpha }(x,y)$ is a nonlinear function, given by

3 $$\begin{aligned} f_{\alpha }(x,y)= {\left\{ \begin{array}{ll} \frac{\alpha }{(1-x)}+y,\ &{} \ x \le 0\\ \alpha +y,\ &{}\ 0<x<\alpha +y\\ -1,\ &{}\ x\ge \alpha +y, \end{array}\right. } \end{aligned}$$

**Figure 10:**
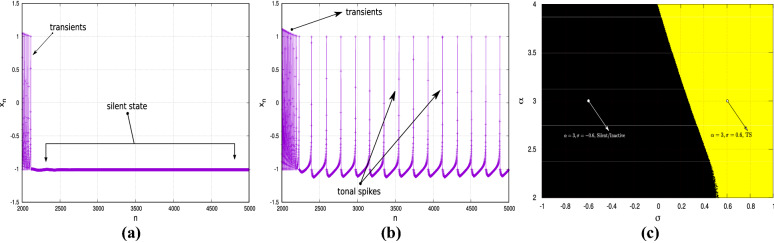
Time-series plots of an isolated neuron exhibiting different dynamical states at long time: (**a**) dead/silent (DS), (**b**) tonal spiking (TS). (**c**) The dynamical state in the $(\alpha ,\sigma )$ parameter space: black is the DS state; yellow is the TS state. The arrow-marked points in (**c**) correspond to $\alpha =3,\sigma =\pm 0.6$ and are the values of these parameters used in the numerical examples.

$\alpha$ and $\mu$ are time-independent parameters that defines the qualitative nature of the dynamics of an isolated neuron, while $\beta _{n}$ and $\sigma _{n}$ are time dependent parameters that describe the external influences (like injected current) to the system. The spiking frequency increases with $\sigma _n$^29^. For a neuron in isolation, these parameters can be treated as constants and can be simplified by putting $\beta _{n}=0$ and $\sigma _{n}=\sigma \ \forall \ n$. Depending on the choice of the parameters, a solitary neuron exhibits three distinct long-time dynamical states^29^: dead/silent (DS), tonal spiking (TS) and spiking bursting (SB). In DS state, the isolated neurons show no activity; see Fig. 10a. The TS state is characterised by the neurons showing continuous spikes; see Fig. 10b.

In the SB state, the neuron shows bursts of spikes, which may be chaotic depending on the parameter values. The parameter regime considered in this study is limited to the DS and TS states and can be seen in Fig. 10c; here, the black region is the DS state while the yellow region denotes the TS state. Beyond $\alpha =4$, there exists the SB regime which is not depicted here because it is not relevant to this study.

### Network of neurons

For a network of coupled Rulkov neurons, Eqs. (1–2) are written in the modified form as

4 $$\begin{aligned} x_{i,n+1}&=f_{\alpha _{i}}(x_{i,n},\ y_{i,n}+\beta _{i,n}), \end{aligned}$$

5 $$\begin{aligned} y_{i,n+1}&=y_{i,n}-\mu (x_{i,n}+1)+\mu (\sigma _{i}+\sigma _{i,n}), \end{aligned}$$

where

6a $$\begin{aligned} \sigma _{i,n}= & {} {\left\{ \begin{array}{ll} \frac{1}{k_{i}}\sum _{j\ne i}\Big [g_{ij,n}\sigma ^{e}(x_{j,n}-x_{i,n})\Big ]\ &{} \mathrm{if}\,\,\, k_i \ne 0\\ 0 &{} \mathrm{if}\,\,\, k_i = 0 \end{array}\right. } \end{aligned}$$

6b $$\begin{aligned} \beta _{i,n}= & {} {\left\{ \begin{array}{ll} \frac{1}{k_{i}}\sum _{j\ne i}\Big [g_{ij,n}\beta ^{e}(x_{j,n}-x_{i,n})\Big ]\ &{} \mathrm{if}\,\,\, k_i \ne 0 \\ 0 &{} \mathrm{if}\,\,\, k_i = 0 \end{array}\right. } \end{aligned}$$

for all $i,j=1,\ldots ,N$. Here, the suffices *i*, *j* denote the neuron number, *N* denotes the total number of neurons in the network, the parameters $\alpha _{i}$ and $\sigma _{i}$ define the individual dynamical nature of the $i$th neuron,

7 $$\begin{aligned} k_{i}=\sum _{j=1}^{N}G_{ij} \end{aligned}$$

is the degree of the *i*th node indicating the number of connections of a particular neuron, $G_{ij}$ is an element of the $N\times N$ adjacency matrix of the underlying network, $g_{ij,n}$ is the time dependent coupling strength between the *i*-th and the *j*-the neuron at time instant *n* and $\sigma ^e$ and $\beta ^e$ denote the coupling constants. When $\sigma ^{e}=1$ and $\beta ^{e}=1$ (which implies that $\sigma _{i,n}=\beta _{i,n}\ \forall \ i,n$), the model reduces to the original coupling model discussed by Rulkov^29^ for $N=2$. This also implies a symmetric coupling with respect to both $x_{i,n}$ and $y_{i,n}$. This is a mean-field type coupling which is evident from the fact that the coupling term is a mean of the differences between the membrane potential $x_{i,n}$ of the *i*-th neuron and the corresponding membrane potentials $x_{j,n}$ of all other neurons it is directly connected to, weighted with a coupling strength $g_{ij,n}$. Non-symmetric couplings can also be generated by choosing different values for $\sigma ^{e}$ and $\beta ^{e}$.

The most general form of the time-dependent coupling strength $g_{ij,n}$ is given by

8 $$\begin{aligned} g_{ij,n}=\Big [g_{m} + D\zeta _{n}\Big ]G_{ij}, \end{aligned}$$

where $g_{m}$ is the global mean coupling strength, $\zeta _{n}$ represents the discretized white noise process at iterate *n* and *D* is the noise intensity. For the noise-free case, $D=0$, which implies

9 $$\begin{aligned} \beta _{i,n}=\sigma _{i,n}={\left\{ \begin{array}{ll}\frac{g_{m}}{k_{i}}\sum ^{N}_{j\ne i}\Big [G_{ij}(x_{j,n}-x_{i,n})\Big ]\ &{}\,\mathrm{for}\,\, k_{i}\ne 0\\ 0\ &{} \mathrm{for}\,\, k_{i}= 0. \end{array}\right. } \end{aligned}$$

### Order parameter

For a network with given values of $g_m$, *D*, $P_n$—the connection probability of the network—and $P_d$—the probability of a particular neuron being inactive, the collective dynamical state is characterised in terms of an order parameter measure defined as

10 $$\begin{aligned} A \equiv A(P_d;\ g_m,\ P_n,\ D)=\frac{a(P_d) }{\underset{0<P_{d}<1}{\text {max}}[a (P_d)]}, \end{aligned}$$

where, for a given value of $P_d$,

11 $$\begin{aligned} a(P_d)=\frac{1}{N}\sum _{i=1}^{N}\big |\underset{n}{\text {max}}(x_{i,n})-\underset{n}{\text {min}}(x_{i,n})\big | \end{aligned}$$

defines a measure of the largest amplitude of oscillation averaged across all neurons for specified values of $P_d$, $g_m$, $P_n$ and *D*. Here, $\underset{n}{\text {max}}(x_{i,n})$ and $\underset{n}{\text {min}}(x_{i,n})$ indicates the maximum and minimum of $x_i$—the potential corresponding to the *i*th neuron over all time steps *n*. The denominator in Eq. (10) refers to the maximum value of $a(P_d)$ when $P_d$ is varied from 0 to 1, while $P_n$, $g_m$ and *D* are kept constant. The measure *A* is therefore a normalized average amplitude measure. These order parameters are similar to those studied for similar systems in literature^15^.

### Simulation details

The network structure is taken to be the Erdős–Rényi (ER) random graph with $N=2\times 10^3$ nodes. The connection probability $P_{n}$ is varied from 0.1 to 0.9, thereby covering the whole range of sparse and dense random networks. The initial conditions for each node are assumed to be random and sampled from a uniform distribution between $[-1,1]$. The map is iterated over $8\times 10^3$ iterates. In computing *A*, data from the first $5\times 10^3$ iterates are discarded and only the last $3\times 10^3$ are used. This is carried out to ensure that the transient effects have died down and only the values after stationarity has been achieved are used in computing the quantitate measures used for the analysis. In all the simulations, $\mu$ is set to 0.001 to enable spiking state in the neurons. For the parametric analysis in the $g_m-P_d$ parameter space, a grid is defined by varying $g_{m}$ between [0, 1] in steps of 0.05 and $P_{d}$ is varied between [0, 1] in steps of 0.01. Simulations are carried out for each of these grid points. The noise $D\zeta _{n}$ was generated directly using the $gsl\_ran\_gaussian()$ function from the GNU Scientific Library (GSL), which is an open-source and freely available library. The parametric studies were conducted using a direct grid search type algorithm within the predefined parameter range. All the simulations were performed in *double* precision for accuracy using C++ programs which were further parallelized using OpenMP for improved performance. All the plots were generated using the plotting capabilities of either MATLAB R2020b or gnuplot 5.2.

## Data Availability

Data generated during this study and the computer simulation codes are available upon reasonable request.
